# Thermodynamics Analysis of Multiple Microelements’ Coupling Behavior in High Fatigue Resistance 50CrVA Spring Steel with Nanoparticles

**DOI:** 10.3390/ma12182952

**Published:** 2019-09-11

**Authors:** Yanlin Wang, Lihua Fu, Meng Zhou, Zirong Zhou, Xiaolu Pang, Shouyan Zhong, Alex A. Volinsky

**Affiliations:** 1 School of Materials Science and Engineering, University of Science and Technology Beijing, Beijing 100083, China; Wangyanlin921@aliyun.com; 2 School of Mechanical Engineering, Dongguan University of Technology, Dongguan 523808, China; zhouzirong1234@163.com (Z.Z.); zhongshouyan66@163.com (S.Z.); 3 School of Materials Science and Engineering, Henan University of Science and Technology, Luoyang 471023, China; zhoumeng0902@126.com; 4 Department of Mechanical Engineering, University of South Florida, Tampa, FL 33620, USA; volinsky@usf.edu

**Keywords:** multiple microelements, thermodynamics analysis, nanoparticles, fatigue behavior, spring steel

## Abstract

Solid solution and coupling precipitation behavior of multiple microelements in 50CrVA spring steel under different temperatures were analyzed based on thermodynamics. Quantitative relationships between the multiple microelements’ contents and secondary phases, and their effects on fatigue life, were systematically studied in conjunction with the secondary phase microstructure characterization using scanning and transmission electron microscopy, etc. The solid solution contents of different microelements decreased as the temperature decreased, especially N and Ti, but the number of compounds gradually increased when the temperature decreased. Carbonitride constitutional liquation occurred in 50CrVA-S1# spring steel-containing microparticles, and without carbonitrides, constitutional liquation occurred in 50CrVA-S2# spring steel-containing nanoparticles. The experimental results indicate that the fatigue life reduces by about an order of magnitude when the secondary phase size changes from nanometers to microns, and the corresponding relationship among multiple microelements, microstructure of secondary phases, and fatigue life, was established in this spring steel.

## 1. Introduction

The precision design of materials chemical composition, microstructure and performance is the pursuit goal for materials researchers. This includes multiple microelements and their coupling precipitation behavior, which determine spring steel performance [1]. Spring steel is one of the key equipment components, and its quality improvement is a prerequisite to guarantee adequate performance [2,3]. Demand for high performance spring steel is increasing due to improvements in automobile lightweight and safety, which further demand longer service life [4]. 50CrVA spring steel is one of the more common automobile components prone to fatigue failures, accounting for 50% of the major failure modes and as much as 90% in some extreme cases [5]. The research shows that fatigue failure can be attributed to many factors, such as microstructure, stress ratio, inclusion size, location, etc. [6,7,8]. These inclusions come from deoxidation additions or impurities at the subsurface level, which are the locations for fatigue crack nucleation when gigacycle fatigue levels are reached [9]. These cracks will continue to propagate, eventually leading to fatigue failure, which occurs instantaneously without any warning. Thus, fatigue failure of steel springs has a serious impact on mechanical systems [10,11], even causing serious accidents that endanger human life.

Microstructure, distributed control, and modification of secondary phases in steel play a very important role in improving product quality [12,13]. Specifically, control of secondary phases has become the most important research direction for improving fatigue life of high strength steel. We know that no matter what type of secondary phase is generated by the chemical reactions among the multiple microelements in steels, the scientific design of microelements has a very important direct effect on the secondary phase microstructure. Solid solution and coupling precipitation behavior of multiple microelements in steel have received widespread attention, and thermodynamics analysis and its engineering applications has been a hot research topic for many years [14,15,16]. Unary or binary secondary phase changes in solid solution with temperature can be determined experimentally. The thermodynamics of ternary secondary phases have been studied by many scholars, and the corresponding thermodynamic software has been developed (such as Thermo-Calc) [17,18,19,20]. However, for more multiple secondary phases, it is assumed that the solid solution of a microelement tends towards zero within a certain temperature range in most cases; thus, the calculation is simplified [21,22]. In our previous work [23], based on the coupling mechanism of microelements in Fe–Ti–O system molten steels, the precipitation behavior and their effects on the microstructure of Ti_3_O_5_ particles were investigated, and the formation mechanism of in situ Ti_3_O_5_ nanoparticles in molten steel were also discussed. Here, a thermodynamics analysis method of the Fe–V–Ti–N–C system steel has been developed. It facilitates the calculation of the chemical composition for the multiple secondary phases and relative amounts as a function of spring steel composition and temperature. The quantitative relationship between the content of microelements and secondary phases in 50CrVA spring steel was studied. The solid solution and coupling precipitation behavior of microelements at different temperatures was analyzed. The effects of the microstructure of secondary phases on fatigue life were also studied, and the corresponding relationship among microelements, microstructure of secondary phases and fatigue life in spring steel was established. Furthermore, the microstructure of the 50CrVA spring steel was characterized in this work using scanning and transmission electron microscopy (SEM, TEM), etc.

## 2. Experimental Procedures

### 2.1. Materials

The 50CrVA spring steel developed by FangDa Special Steel Technology Co., Ltd (Nanchang, China) is widely used in automotive springs, and its chemical compositions are shown in Table 1. The steel was fabricated through converter smelting, ladle furnace (LF) refining, vacuum refining processing, continuous casting combined with electromagnetic stirring technique, and continuous rolling and testing processes. The size of the rolling slab was about 180 × 180 × 7950 mm^3^. The initial rolling temperature was about 1050 °C, after which the steel was rolled according to required product specifications (90 × 22 mm^2^), via rough, middle, and finishing rolling procedures, respectively. The finishing rolling temperature was about 950 °C. The steel was air cooled to room temperature.

### 2.2. Fe–V–Ti–N–C System Thermodynamics Calculation Methods

**Figure 1:**
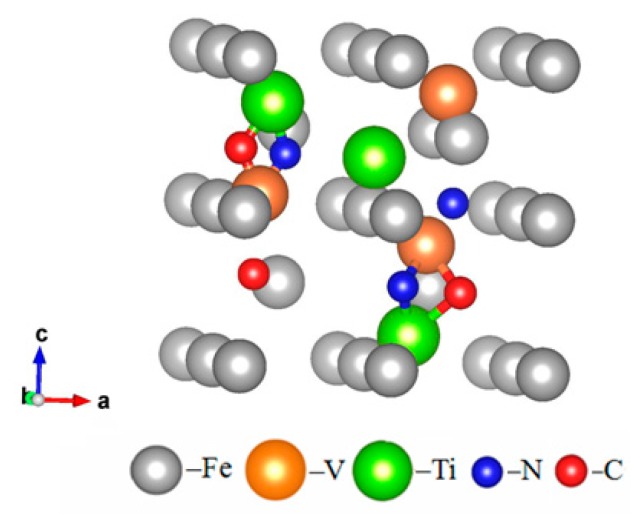
Atomic model of the Fe–V–Ti–N–C system coupling precipitation.

The microelements in steel mainly form a solid solution in the iron matrix and contribute to the formation of the corresponding compound. It is known that different forms have different effects. The atomic model of the Fe–V–Ti–N–C system coupling precipitation as shown in Figure 1, and the compounds formed at different temperature also have different precipitation characteristics and effects [22].

For the Fe–V–Ti–N–C system, the microalloying elements can react with the C or N elements to form multiple secondary phases. These secondary phases are mainly composed of carbides and nitrides, which have similar crystal structure and possess continuous or extended mutual solubility. Therefore, these multiple secondary phases can be expressed as the same chemical formula (V, Ti) (Ti) (C, N). The valid concentrations of microalloying elements V and Ti as well as interstitial elements C and N in these systems, and their activities obey Henry’s law. In this case, the effective activity coefficients of the VC, TiC, VN, and TiN materials are assumed to be *k*_1_, *k*_2_, *m*_1_, and *m*_2_, respectively. Therefore, the carbonitrides can be written as V_(*k*_1_+*m*_1_)_Ti_(*k*_2_+*m*_2_)_C_(*k*_1_+*k*_2_)_N_(*m*_1_+*m*_2_)_. Then, it is assumed that the moles amount of the carbonitride V_(*k*_1_+*m*_1_)_Ti_(*k*_2_+*m*_2_)_C_(*k*_1_+*k*_2_)_N_(*m*_1_+*m*_2_)_ in the steel is *t* moles. The carbonitride can be viewed as a mixture of pure binary carbides (VC, TiC) and nitrides (VN, TiN). The moles amount of the VC, TiC, VN, and TiN will be *k*_1_*t*, *k*_2_*t*, m_1_*t*, and m_2_*t*, respectively. Combining Wagner’s formalism [24] with the mass conservation, the composition of the matrix phase and the precipiate phase and the total moles amount of the precipitate for the carbonitride V_(*k*_1_+*m*_1_)_Ti_(*k*_2_+*m*_2_)_C_(*k*_1_+*k*_2_)_N_(*m*_1_+*m*_2_)_ under appropriate temperature can be calculated on thermodynamics.

#### 2.2.1. Wagner’s Formalism

The chemical reaction among V, Ti, C, and N in steels and its equilibrium constant are given by Equations (1) and (2) in the presence of solid V_(*k*_1_+*m*_1_)_Ti_(*k*_2_+*m*_2_)_C_(*k*_1_+*k*_2_)_N_(*m*_1_+*m*_2_)_:

(1) $$tV_{(}{{}_{k}}_{{}_{1}+}{{}_{m}}_{{}_{1})}{\mathrm{Ti}}_{(}{{}_{k}}_{{}_{2}+}{{}_{m}}_{{}_{2})}C_{(}{{}_{k}}_{{}_{1}+}{{}_{k}}_{{}_{2})}N_{(}{{}_{m}}_{{}_{1}+}{{}_{m}}_{{}_{2})}=(k_{1}+m_{1})tV+(k_{2}+m_{2})t\mathrm{Ti}+(k_{1}+k_{2})tC+(m_{1}+m_{2})tN$$

i.e.,

V_(*k*_1_+*m*_1_)_Ti_(*k*_2_+*m*_2_)_C_(*k*_1_+*k*_2_)_N_(*m*_1_+*m*_2_)_ = (*k*_1_+*m*_1_)V + (*k*_2_+*m*_2_)Ti + (*k*_1_+*k*_2_)C + (*m*_1_+*m*_2_)N

Therefore, the reaction equilibrium constant can be expressed as:

(2) $$K_{\mathrm{VI}}^{\theta }=\frac{h_{V}^{{}^{k_{{}_{1}}+m_{{}_{1}}}}\cdot h_{\mathrm{Ti}}^{{}^{k_{{}_{2}}+m_{{}_{2}}}}\cdot h_{C}^{{}^{k_{{}_{1}}+K_{{}_{2}}}}\cdot h_{N}^{{}^{m_{{}_{1}}+m_{{}_{2}}}}}{a_{V_{(}{{}_{k}}_{{}_{1}+}{{}_{m}}_{{}_{1})}{\mathrm{Ti}}_{(}{{}_{k}}_{{}_{2}+}{{}_{m}}_{{}_{2})}C_{(}{{}_{k}}_{{}_{1}+}{{}_{k}}_{{}_{2})}N_{(}{{}_{m}}_{{}_{1}+}{{}_{m}}_{{}_{2})}(s)}}=\frac{f_{V}^{k_{1}+m_{1}}V^{k_{1}+m_{1}}\cdot f_{\mathrm{Ti}}^{k_{2}+m_{2}}{\mathrm{Ti}}^{k_{2}+m_{2}}\cdot f_{C}^{k_{1}+k_{2}}C^{k_{1}+k_{2}}\cdot f_{N}^{m_{1}+m_{2}}N^{m_{1}+m_{2}}}{a_{V_{(}{{}_{k}}_{{}_{1}+}{{}_{m}}_{{}_{1})}{\mathrm{Ti}}_{(}{{}_{k}}_{{}_{2}+}{{}_{m}}_{{}_{2})}C_{(}{{}_{k}}_{{}_{1}+}{{}_{k}}_{{}_{2})}N_{(}{{}_{m}}_{{}_{1}+}{{}_{m}}_{{}_{2})}(s)}}$$

where *K*, *a*, *h*, *f*, and [*i*] denote the equilibrium constant, the Raoult activity, the Henry activity, the Henrian activity coefficient, and concentration of *i* in steel (mass %), respectively.

Since the overall reaction can be seen as consisting of the following subreactions:

V_(*k*_1_+*m*_1_)_Ti_(*k*_2_+*m*_2_)_C_(*k*_1_+*k*_2_)_N_(*m*_1_+*m*_2_)_ = *k*_1_[VC] + *k*_2_[TiC] + *m*_1_[VN] + *m*_2_[TiN] (3)

*k*_1_[VC] = *k*_1_[V] + *k*_1_[C] (4)

*k*_2_[TiC] = *k*_2_[Ti] + *k*_2_[C] (5)

*m*_1_[VN] = *m*_1_[V] + *m*_1_[N] (6)

*m*_2_[TiN] = *m*_2_[Ti] + *m*_2_[N] (7)

Therefore, the corresponding reaction equilibrium constants can be obtained by:

(8) $$K_{I}^{\theta }=\frac{h_{\mathrm{VC}}^{{}^{k_{1}}}\cdot h_{\mathrm{TiC}}^{{}^{k_{2}}}\cdot h_{\mathrm{VN}}^{{}^{m_{1}}}\cdot h_{\mathrm{TiN}}^{{}^{m_{2}}}}{a_{V_{(}{{}_{k}}_{1+}{{}_{m}}_{1)}{\mathrm{Ti}}_{(}{{}_{k}}_{2+}{{}_{m}}_{2)}C_{(}{{}_{k}}_{1+}{{}_{k}}_{2)}N_{(}{{}_{m}}_{1+}{{}_{m}}_{2)}(s)}}=\frac{f_{\mathrm{VC}}^{k_{1}}{\left[\mathrm{VC}\right]}^{k_{1}}\cdot f_{\mathrm{TiC}}^{k_{2}}{\left[\mathrm{TiC}\right]}^{k_{2}}\cdot f_{\mathrm{VN}}^{m_{1}}{\left[\mathrm{VN}\right]}^{m_{1}}\cdot f_{\mathrm{TiN}}^{m_{2}}{\left[\mathrm{TiN}\right]}^{m_{2}}}{a_{V_{(}{{}_{k}}_{1+}{{}_{m}}_{1)}{\mathrm{Ti}}_{(}{{}_{k}}_{2+}{{}_{m}}_{2)}C_{(}{{}_{k}}_{1+}{{}_{k}}_{2)}N_{(}{{}_{m}}_{1+}{{}_{m}}_{2)}(s)}}$$

Here, *k*_1_, *k*_2_, *m*_1_, and *m*_2_ denote the effective activity coefficients of the components VC, TiC, VN, and TiN, respectively.

From *k*_1_[VC] = *k*_1_[V] + *k*_1_[C], i.e.:

[VC] = [V] + [C](9)

Therefore:

(10) $$K_{\mathrm{VC}}=(h_{V}^{}\cdot h_{C}^{})/a_{\mathrm{VC}(s)}=\left\{f_{V}^{}{\left[V\right]}^{}\cdot f_{C}^{}{\left[C\right]}^{}\right\}/a_{\mathrm{VC}(s)}$$

The standard states of $h_{V}^{}$ and $h_{C}^{}$ in Equation (10) are infinitely dilute solutions for V and C in steels. Taking the logarithm of both sides and rearrangement of Equation (10) gives Equation (11).

(11) $$\mathrm{lg}K_{\mathrm{VC}}=\mathrm{lg}f_{V}+\mathrm{lg}f_{C}+\mathrm{lg}[V]+\mathrm{lg}[C]-\mathrm{lg}a_{\mathrm{VC}(s)}$$

Each activity coefficient in Equation (11) can be expressed by Equations (12) and (13).

(12) $$\mathrm{lg}f_{V}=\sum_{i=1}^{n}e_{V}^{i}[i]+\sum_{i=1}^{n}r_{V}^{i}{\left[i\right]}^{2}+\sum_{i=1}^{n}r_{V}^{i,V}[i][V]$$

(13) $$\mathrm{lg}f_{C}=\sum_{i=1}^{n}e_{C}^{i}[i]+\sum_{i=1}^{n}r_{C}^{i}{\left[i\right]}^{2}+\sum_{i=1}^{n}r_{C}^{i,C}[i][C]$$

where $e_{i}^{j}$, $r_{i}^{j}$ and $r_{i}^{j,i}$ denote the first order, second order interaction parameters between *i* and *j*, and cross-product term, respectively. It was assumed that second order parameter and cross-product term could be ignored in the present work, and $a_{\mathrm{VC}(s)}$ was *k*_1_.

Therefore:

(14) $$\mathrm{lg}\left\{\left[V\right]\left[C\right]\right\}=\mathrm{lg}K_{\mathrm{VC}}-\sum_{i=1}^{n}e_{V}^{i}[i]-\sum_{i=1}^{n}r_{V}^{i}{\left[i\right]}^{2}-\sum_{i=1}^{n}r_{V}^{i,V}[i][V]-\sum_{i=1}^{n}e_{C}^{i}[i]-\sum_{i=1}^{n}r_{C}^{i}{\left[i\right]}^{2}-\sum_{i=1}^{n}r_{C}^{i,C}[i][C]+\mathrm{lg}k_{1}$$

i.e.:

(15) $$\mathrm{lg}\left\{\left[V\right]\left[C\right]\right\}-\mathrm{lg}k_{1}=\mathrm{lg}K_{\mathrm{VC}}-\sum_{i=1}^{n}e_{V}^{i}[i]-\sum_{i=1}^{n}r_{V}^{i}{\left[i\right]}^{2}-\sum_{i=1}^{n}r_{V}^{i,V}[i][V]-\sum_{i=1}^{n}e_{C}^{i}[i]-\sum_{i=1}^{n}r_{C}^{i}{\left[i\right]}^{2}-\sum_{i=1}^{n}r_{C}^{i,C}[i][C]$$

Then:

(16) $$\mathrm{lg}\left\{\frac{\left[V\right]\left[C\right]}{k_{1}}\right\}=\mathrm{lg}K_{\mathrm{VC}}-\sum_{i=1}^{n}e_{V}^{i}[i]-\sum_{i=1}^{n}r_{V}^{i}{\left[i\right]}^{2}-\sum_{i=1}^{n}r_{V}^{i,V}[i][V]-\sum_{i=1}^{n}e_{C}^{i}[i]-\sum_{i=1}^{n}r_{C}^{i}{\left[i\right]}^{2}-\sum_{i=1}^{n}r_{C}^{i,C}[i][C]$$

For the same reason, we have:

(17) $$\mathrm{lg}\left\{\frac{\left[\mathrm{Ti}\right]\left[C\right]}{k_{2}}\right\}=\mathrm{lg}K_{\mathrm{TiC}}-\sum_{i=1}^{n}e_{\mathrm{Ti}}^{i}[i]-\sum_{i=1}^{n}r_{\mathrm{Ti}}^{i}{\left[i\right]}^{2}-\sum_{i=1}^{n}r_{\mathrm{Ti}}^{i,\mathrm{Ti}}[i][\mathrm{Ti}]-\sum_{i=1}^{n}e_{C}^{i}[i]-\sum_{i=1}^{n}r_{C}^{i}{\left[i\right]}^{2}-\sum_{i=1}^{n}r_{C}^{i,C}[i][C]$$

(18) $$\mathrm{lg}\left\{\frac{\left[V\right]\left[N\right]}{m_{1}}\right\}=\mathrm{lg}K_{\mathrm{VN}}-\sum_{i=1}^{n}e_{V}^{i}[i]-\sum_{i=1}^{n}r_{V}^{i}{\left[i\right]}^{2}-\sum_{i=1}^{n}r_{V}^{i,V}[i][V]-\sum_{i=1}^{n}e_{N}^{i}[i]-\sum_{i=1}^{n}r_{N}^{i}{\left[i\right]}^{2}-\sum_{i=1}^{n}r_{N}^{i,N}[i][N]$$

(19) $$\mathrm{lg}\left\{\frac{\left[\mathrm{Ti}\right]\left[N\right]}{m_{2}}\right\}=\mathrm{lg}K_{\mathrm{TiN}}-\sum_{i=1}^{n}e_{\mathrm{Ti}}^{i}[i]-\sum_{i=1}^{n}r_{\mathrm{Ti}}^{i}{\left[i\right]}^{2}-\sum_{i=1}^{n}r_{\mathrm{Ti}}^{i,\mathrm{Ti}}[i][\mathrm{Ti}]-\sum_{i=1}^{n}e_{N}^{i}[i]-\sum_{i=1}^{n}r_{N}^{i}{\left[i\right]}^{2}-\sum_{i=1}^{n}r_{N}^{i,N}[i][N]$$

Therefore, the content (mass %) of V, Ti, C, and N in steels are *V*, *Ti*, *C*, and *N*, respectively, and the interaction among V, Ti, C, and N in the solid solution state must conform to Wagner’s formalism; thus, we obtain:

(20) $$\mathrm{lg}\left\{\frac{\left[V\right]\left[C\right]}{k_{1}}\right\}=\mathrm{lg}K_{\mathrm{VC}}-\sum_{i=1}^{n}e_{V}^{i}[i]-\sum_{i=1}^{n}r_{V}^{i}{\left[i\right]}^{2}-\sum_{i=1}^{n}r_{V}^{i,V}[i][V]-\sum_{i=1}^{n}e_{C}^{i}[i]-\sum_{i=1}^{n}r_{C}^{i}{\left[i\right]}^{2}-\sum_{i=1}^{n}r_{C}^{i,C}[i][C]$$

(21) $$\mathrm{lg}\left\{\frac{\left[\mathrm{Ti}\right]\left[C\right]}{k_{2}}\right\}=\mathrm{lg}K_{\mathrm{TiC}}-\sum_{i=1}^{n}e_{\mathrm{Ti}}^{i}[i]-\sum_{i=1}^{n}r_{\mathrm{Ti}}^{i}{\left[i\right]}^{2}-\sum_{i=1}^{n}r_{\mathrm{Ti}}^{i,\mathrm{Ti}}[i][\mathrm{Ti}]-\sum_{i=1}^{n}e_{C}^{i}[i]-\sum_{i=1}^{n}r_{C}^{i}{\left[i\right]}^{2}-\sum_{i=1}^{n}r_{C}^{i,C}[i][C]$$

(22) $$\mathrm{lg}\left\{\frac{\left[V\right]\left[N\right]}{m_{1}}\right\}=\mathrm{lg}K_{\mathrm{VN}}-\sum_{i=1}^{n}e_{V}^{i}[i]-\sum_{i=1}^{n}r_{V}^{i}{\left[i\right]}^{2}-\sum_{i=1}^{n}r_{V}^{i,V}[i][V]-\sum_{i=1}^{n}e_{N}^{i}[i]-\sum_{i=1}^{n}r_{N}^{i}{\left[i\right]}^{2}-\sum_{i=1}^{n}r_{N}^{i,N}[i][N]$$

(23) $$\mathrm{lg}\left\{\frac{\left[\mathrm{Ti}\right]\left[N\right]}{m_{2}}\right\}=\mathrm{lg}K_{\mathrm{TiN}}-\sum_{i=1}^{n}e_{\mathrm{Ti}}^{i}[i]-\sum_{i=1}^{n}r_{\mathrm{Ti}}^{i}{\left[i\right]}^{2}-\sum_{i=1}^{n}r_{\mathrm{Ti}}^{i,\mathrm{Ti}}[i][\mathrm{Ti}]-\sum_{i=1}^{n}e_{N}^{i}[i]-\sum_{i=1}^{n}r_{N}^{i}{\left[i\right]}^{2}-\sum_{i=1}^{n}r_{N}^{i,N}[i][N]$$

(24) $$k_{1}+k_{2}+m_{1}+m_{2}=1$$

where [V], [Ti], [C], and [N] are the concentrations (in wt%) of the respective elements dissolved in the solution, respectively. The main interaction parameters of this product used for calculation are shown in Table 2.

#### 2.2.2. Mass Conservation

Furthermore, the addition of each microelement into the steel must conform to the law of mass conservation, and the total composition of each microelement is constant when forming a solid solution or compound. Therefore:

(25) $$\frac{V}{A_{V}}-\frac{\left[V\right]}{A_{V}}=\left(k_{1}+m_{1}\right)t$$

(26) $$\frac{Ti}{A_{\mathrm{Ti}}}-\frac{\left[\mathrm{Ti}\right]}{A_{Ti}}=\left(k_{2}+m_{2}\right)t$$

(27) $$\frac{C}{A_{C}}-\frac{\left[C\right]}{A_{C}}=(k_{1}+k_{2})t$$

(28) $$\frac{N}{A_{N}}-\frac{\left[N\right]}{A_{N}}=(m_{1}+m_{2})t$$

Then, Equation (29) can be acquired by combining Equations (25)–(28):

(29) $$\frac{V}{A_{V}}+\frac{Ti}{A_{\mathrm{Ti}}}+\frac{C}{A_{C}}+\frac{N}{A_{N}}=\frac{\left[V\right]}{A_{V}}+\frac{\left[\mathrm{Ti}\right]}{A_{\mathrm{Ti}}}+\frac{\left[C\right]}{A_{C}}+\frac{\left[N\right]}{A_{N}}+2t$$

where A_V_, A_Ti_, A_C_, and A_N_ are the atomic weights of V, Ti, C, and N, respectively.

Therefore, the thermodynamics calculation model of coupling behavior for the multiple microelements in the Fe–V–Ti–N–C system microalloyed steel have been developed, and we get:

(30) $$\mathrm{lg}\left\{\frac{\left[V\right]\left[C\right]}{k_{1}}\right\}=\mathrm{lg}K_{\mathrm{VC}}-\sum_{i=1}^{n}e_{V}^{i}[i]-\sum_{i=1}^{n}r_{V}^{i}{\left[i\right]}^{2}-\sum_{i=1}^{n}r_{V}^{i,V}[i][V]-\sum_{i=1}^{n}e_{C}^{i}[i]-\sum_{i=1}^{n}r_{C}^{i}{\left[i\right]}^{2}-\sum_{i=1}^{n}r_{C}^{i,C}[i][C]$$

(31) $$\mathrm{lg}\left\{\frac{\left[\mathrm{Ti}\right]\left[C\right]}{k_{2}}\right\}=\mathrm{lg}K_{\mathrm{TiC}}-\sum_{i=1}^{n}e_{\mathrm{Ti}}^{i}[i]-\sum_{i=1}^{n}r_{\mathrm{Ti}}^{i}{\left[i\right]}^{2}-\sum_{i=1}^{n}r_{\mathrm{Ti}}^{i,\mathrm{Ti}}[i][\mathrm{Ti}]-\sum_{i=1}^{n}e_{C}^{i}[i]-\sum_{i=1}^{n}r_{C}^{i}{\left[i\right]}^{2}-\sum_{i=1}^{n}r_{C}^{i,C}[i][C]$$

(32) $$\mathrm{lg}\left\{\frac{\left[V\right]\left[N\right]}{m_{1}}\right\}=\mathrm{lg}K_{\mathrm{VN}}-\sum_{i=1}^{n}e_{V}^{i}[i]-\sum_{i=1}^{n}r_{V}^{i}{\left[i\right]}^{2}-\sum_{i=1}^{n}r_{V}^{i,V}[i][V]-\sum_{i=1}^{n}e_{N}^{i}[i]-\sum_{i=1}^{n}r_{N}^{i}{\left[i\right]}^{2}-\sum_{i=1}^{n}r_{N}^{i,N}[i][N]$$

(33) $$\mathrm{lg}\left\{\frac{\left[\mathrm{Ti}\right]\left[N\right]}{m_{2}}\right\}=\mathrm{lg}K_{\mathrm{TiN}}-\sum_{i=1}^{n}e_{\mathrm{Ti}}^{i}[i]-\sum_{i=1}^{n}r_{\mathrm{Ti}}^{i}{\left[i\right]}^{2}-\sum_{i=1}^{n}r_{\mathrm{Ti}}^{i,\mathrm{Ti}}[i][\mathrm{Ti}]-\sum_{i=1}^{n}e_{N}^{i}[i]-\sum_{i=1}^{n}r_{N}^{i}{\left[i\right]}^{2}-\sum_{i=1}^{n}r_{N}^{i,N}[i][N]$$

(34) $$\frac{V}{A_{V}}-\frac{\left[V\right]}{A_{V}}=\left(k_{1}+m_{1}\right)t$$

(35) $$\frac{Ti}{A_{\mathrm{Ti}}}-\frac{\left[\mathrm{Ti}\right]}{A_{\mathrm{Ti}}}=\left(k_{2}+m_{2}\right)t$$

(36) $$\frac{C}{A_{C}}-\frac{\left[C\right]}{A_{C}}=(k_{1}+k_{2})t$$

(37) $$\frac{V}{A_{V}}+\frac{Ti}{A_{\mathrm{Ti}}}+\frac{C}{A_{C}}+\frac{N}{A_{N}}=\frac{\left[V\right]}{A_{V}}+\frac{\left[\mathrm{Ti}\right]}{A_{\mathrm{Ti}}}+\frac{\left[C\right]}{A_{C}}+\frac{\left[N\right]}{A_{N}}+2t$$

(38) $$k_{1}+k_{2}+m_{1}+m_{2}=1$$

Here, A_V_ = 50.9, A_Ti_ = 47.9, A_N_ = 14, and A_C_ = 12. It was assumed that the second order parameter and cross-product term could be ignored in the present work. Equations (30) through (38) have nine unknowns (i.e., [V], [Ti], [C], [N], *k*_1_, *k*_2_, *m*_1_, *m*_2_, and *t*), which are solved numerically to determine the equilibrium state. Further, the numerical iteration calculation process based on the fixed-point iteration method was carried out in Matlab 9.0. Additionally [22]:

(39) $$\mathrm{lg}K_{\mathrm{VC}}=6.72-9500/T$$

(40) $$\mathrm{lg}K_{\mathrm{TiC}}=2.75-7000/T$$

(41) $$\mathrm{lg}K_{\mathrm{VN}}=3.63-8700/T$$

(42) $$\mathrm{lg}K_{\mathrm{TiN}}=0.32-8000/T,$$

where *T* is the temperature (K).

### 2.3. Tests

**Figure 2:**
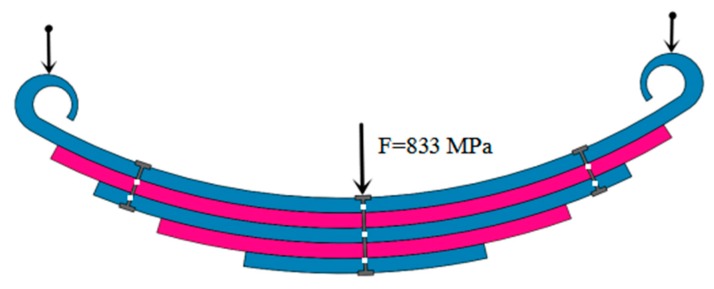
Fatigue tests for automobile plate spring (F = 833 MPa; *f* = 1 Hz).

The microstructure of the steel was further investigated using Zeiss Supra 55 field emission scanning electron microscope (SEM) and JEOL JEM-2100 transmission electron microscope (TEM). The foils for TEM were cut from the steel samples, mechanically thinned to ~35 μm, and then electrochemically polished using a solution of 10 vol.% HClO_4_ methanol electrolyte at a low temperature. Finally, the foils were further ion milled to obtain an electron transparent area. The fatigue properties tests of automobile plate spring were performed using an Instron 8801 fatigue testing system under a frequency of 1 Hz and a stress of 833 MPa. Figure 2 is the schematic diagram of the fatigue tests for automobile plate spring.

## 3. Results and Discussion

### 3.1. Thermodynamics Analysis Results

For the Fe–V–Ti–N–C multiple microelements system in spring steels 50CrVA, the compounds formed are (V, Ti) (C, N). Based on the above thermodynamics analysis model of equilibrium solution for the mutual dissolution and immiscible multiple secondary phases in steels, the equilibrium solution from 800 °C to initial precipitation temperature (such as [V], [Ti], [N], [C], and *t*) for multiple microelements in Fe–V–Ti–N–C system steels were systematically researched. In this formula, [V], [Ti], [N], and [C] are the solid solution contents of V, Ti, N, and C in steels under different temperatures, and *t* is the moles amount of the corresponding compounds [23].

**Figure 3:**
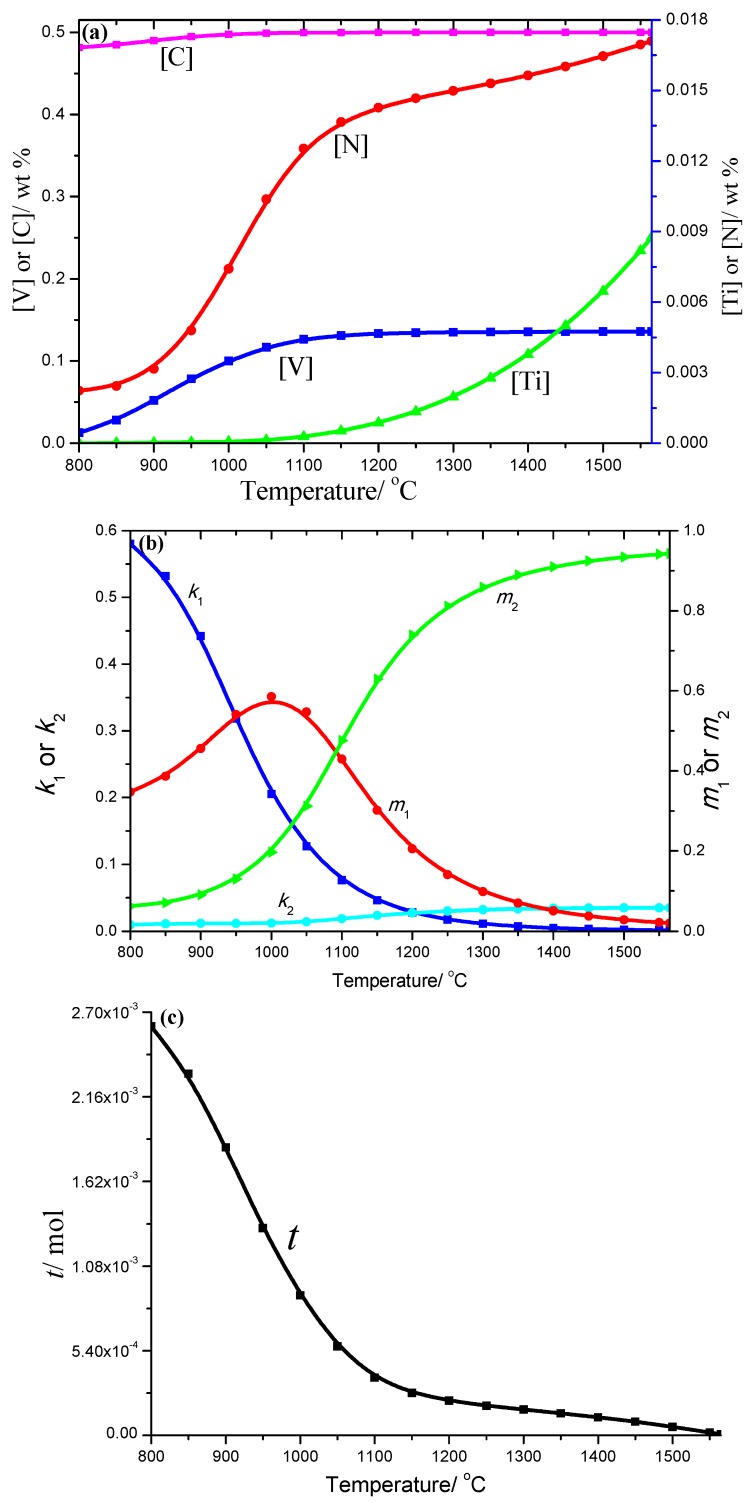
(**a**) Change of solid solution contents; (**b**) *k*_1_, *k*_2_, *m*_1_, *m*_2_ constants and (**c**) total moles number of compounds with temperature obtained from thermodynamics analysis of 50CrVA-S1#.

The 50CrVA-S1# spring steel was thermodynamically analyzed. The results showed that the carbonitrides begin to precipitate at 1565 °C, which is higher than the corresponding liquidus temperature (about 1497 °C). It is known that, for HSLA steels, carbonitrides constitutional liquation occurs when the initial precipitation temperature is above the liquidus temperature. Figure 3 indicates the solid solution contents of different element in this steel, the coefficients like *k*_1_, *k*_2_, *m*_1_, *m*_2_, and the total moles amount (*t*) of the corresponding compounds under different temperatures. It can be seen that the solid solution contents of different elements decreased with the temperature decrease, especially the change of N and Ti, while the change of *k*_1_, *k*_2_, *m*_1_, and *m*_2_ coefficients with temperature was more complex, so the proportion of the compounds precipitated in this steel during the cooling process changed with time. At high temperatures, the proportion of TiN in the quaternary phase precipitates was more obvious, and the proportion of VC in the precipitates was more obvious at low temperatures. The total moles amount (*t*) increased gradually as the temperature decreased, and the [V] is 0.01274%, [Ti] is 4.99 × 10^−8^, [N] is 0.00223%, [C] is 0.48152%, and the total moles amount *t* is 0.00261 mol at 800 °C.

**Figure 4:**
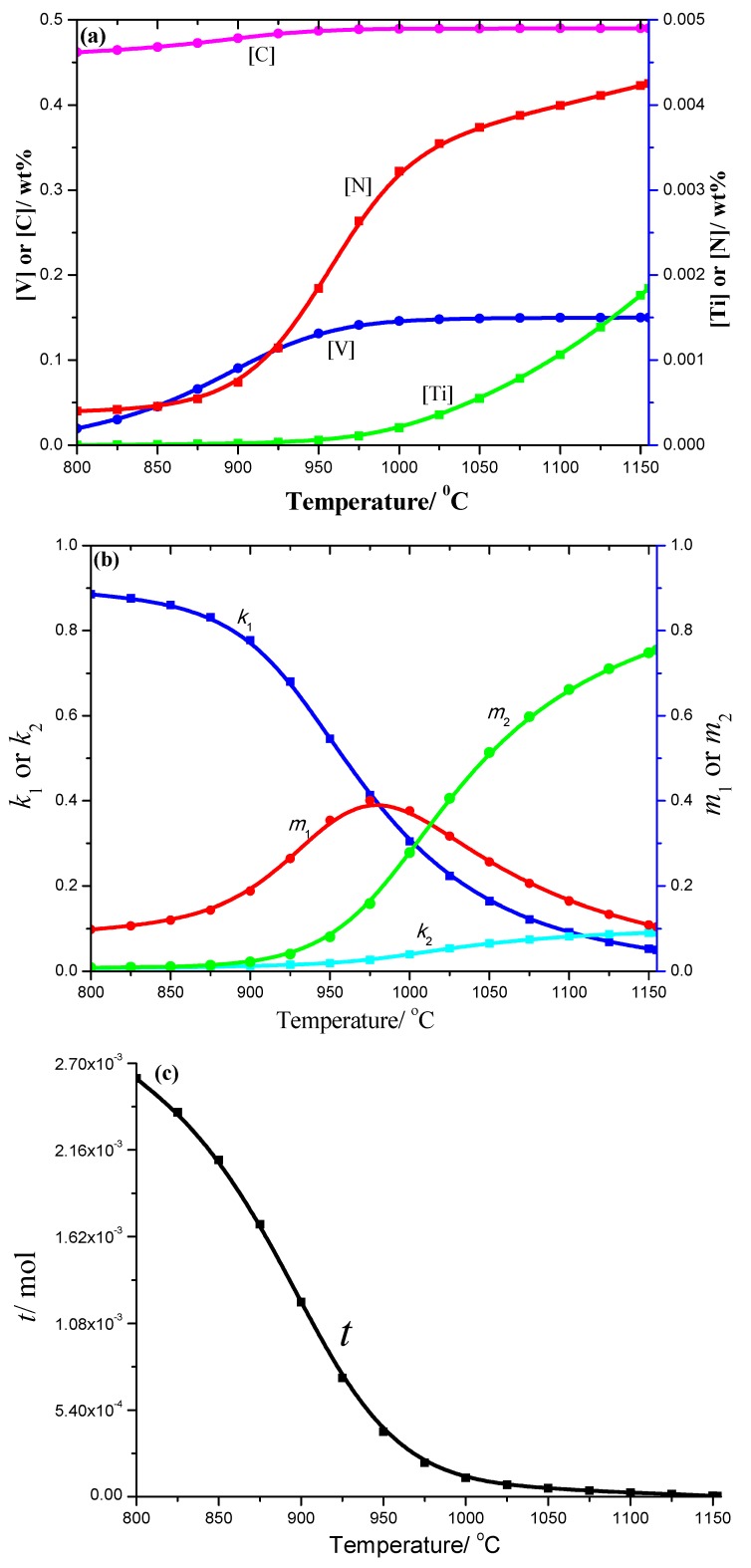
(**a**) Change of solid solution contents; (**b**) *k*_1_, *k*_2_, *m*_1_, *m*_2_ constants and (**c**) total moles number of compounds with temperature obtained from thermodynamics analysis of 50CrVA-S2#.

The 50CrVA-S2# spring steel was also thermodynamically analyzed. The results showed that the carbonitrides begin to precipitate at 1155 °C, which is lower than the corresponding liquidus temperature. Thus, without carbonitrides, constitutional liquation occurred in this steel. The solid solution contents of different elements in this steel, coefficients like *k*_1_, *k*_2_, *m*_1_, *m*_2_, and the total moles amount (*t*) of the corresponding compounds under different temperatures are shown in Figure 4. The solid solution contents of different elements also decreased as the temperature decreased, especially the change of N and Ti. The total moles amount (*t*) of the multiple secondary phase precipitates also increased gradually with decreasing temperature, and the [V] is 0.01948%, [Ti] is 3.65 × 10^−8^, [N] is 4.01 × 10^−6^, [C] is 0.46207%, and the total moles amount *t* is 0.00260 mol at 800 °C.

### 3.2. Secondary Phase Microstructure and Fatigue Life

#### 3.2.1. Secondary Phase Microstructure

**Figure 5:**
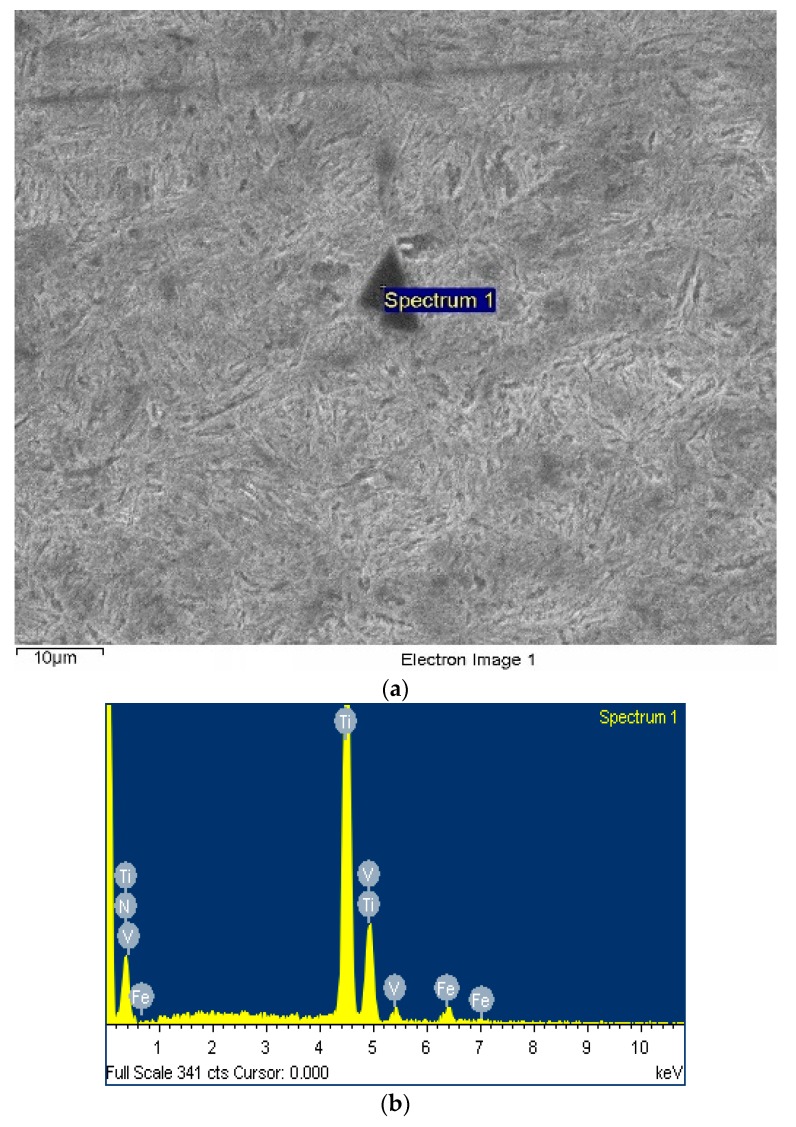
(**a**) The scan results of the secondary phase in 50CrVA-S1# and (**b**) corresponding energy dispersive spectroscopy (EDS) analysis.

**Figure 6:**
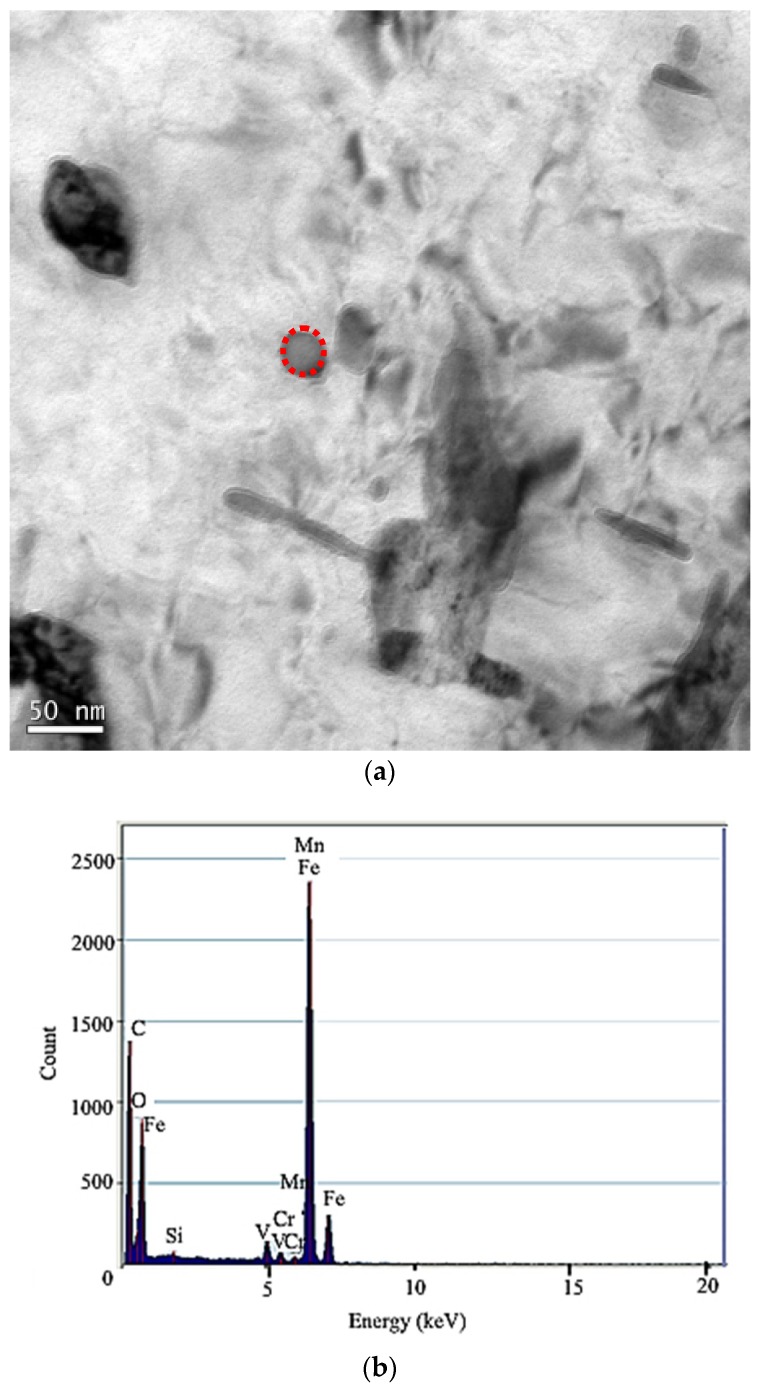
(**a**) TEM image of the secondary phase in 50CrVA-S2# and (**b**) the corresponding EDS analysis.

The microstructure of 50CrVA-S1# spring steel was characterized using a Zeiss Supra 55 field emission scanning electron microscope, shown in Figure 5a. The form of secondary phase is water chestnuts, and the diameter is about 10 µm, which can easily become a source of cracks in fatigue tests. Corresponding energy dispersive spectroscopy (EDS) analysis results of this particle are shown in Figure 5b. The secondary phase composition is mainly (Ti, V)N. The secondary phase precipitation images of 50CrVA-S2# spring steel were obtained using the JEOL JEM-2100 transmission electron microscope, shown in Figure 6a. The diameter of secondary phases in this material is about 10–50 nm. From the corresponding energy dispersive spectroscopy analysis result of the secondary phase in 50CrVA-S2#, shown in Figure 6b, the secondary phase is mainly VC with a diameter of 20 nm.

**Figure 7:**
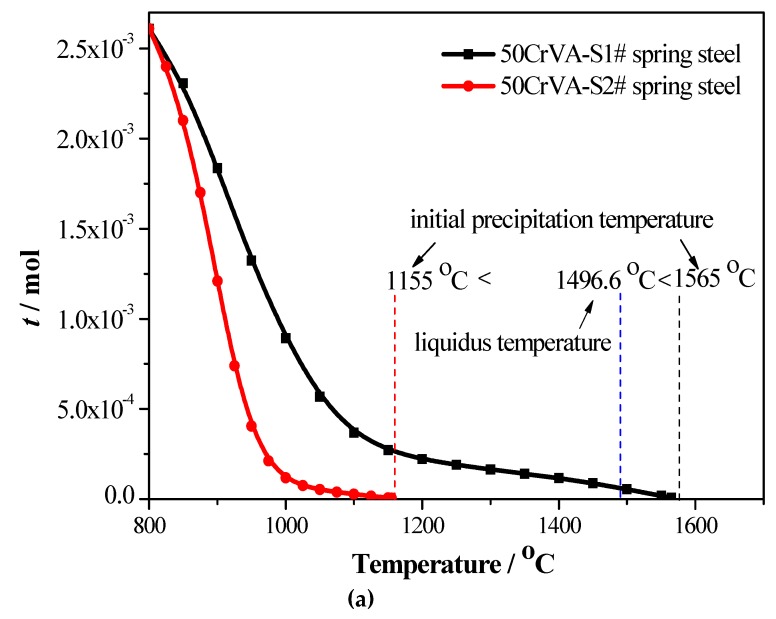

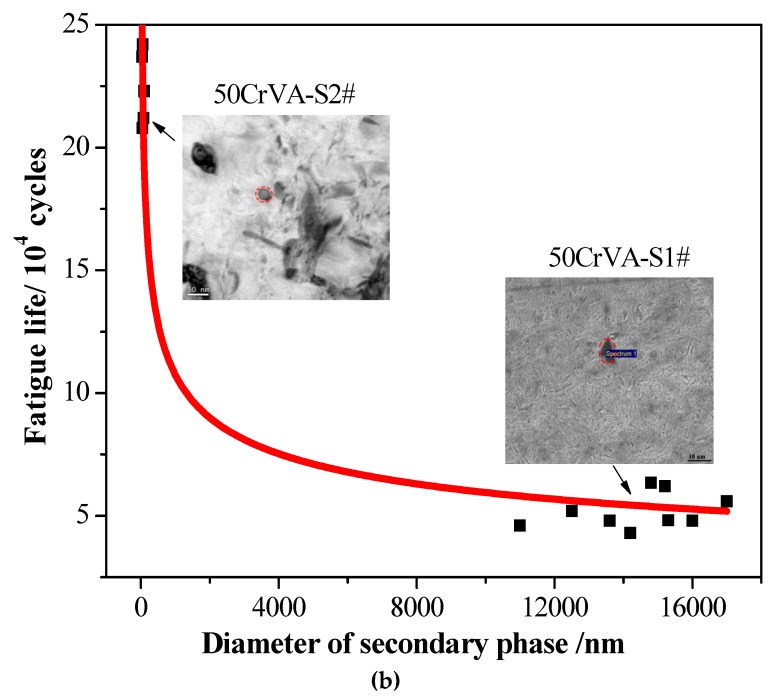
(**a**) The initial precipitation temperature of 50CrVA and (**b**) the relationship between secondary phase size and fatigue life in 50CrVA.

From the thermodynamics analysis results (as shown in Figure 7a), we know that the initial precipitation temperature of 50CrVA-S1# spring steel was 1565 °C, which is higher than the corresponding liquidus temperature of 1496.56 °C for HSLA steels [35]. Thus, it is obvious that strong carbonitrides constitutional liquation has already occurred in 50CrVA-S1#, and the carbonitrides with microparticles are consistent with Figure 5. By contrast, for 50CrVA-S2#, the initial precipitation temperature is 1155 °C (as shown in Figure 7a), which is lower than the liquidus temperature; therefore, without carbonitrides, constitutional liquation occurred in this steel, and the carbonitrides with nanoparticles are consistent with Figure 6.

#### 3.2.2. Fatigue Life

The fatigue life is a key property for spring steel flat bar. The same structure automobile plate springs were prepared using the above two different materials, respectively, and the fatigue property tests were performed using an Instron 8801 fatigue testing system under a frequency of 1 Hz and a stress of 833 MPa. Figure 7b shows the corresponding relationship between the secondary phase size (the maximum secondary phase size detected in fracture place of plate spring) and the fatigue life in 50CrVA spring steel flat bar. The secondary phase size range detected in the fracture surface of 50CrVA-S1# is 10.5–17 µm, and the corresponding fatigue life range is 43–63.5 × 10^3^ cycles. The secondary phase size range detected in the fracture surface of 50CrVA-S2# is 10–100 nm, and the corresponding fatigue life range is 208–242 × 10^3^ cycles. The experimental results indicate that the fatigue life is reduced by about an order of magnitude when the secondary phase size changes from nanometer scale to micron level.

**Figure 8:**
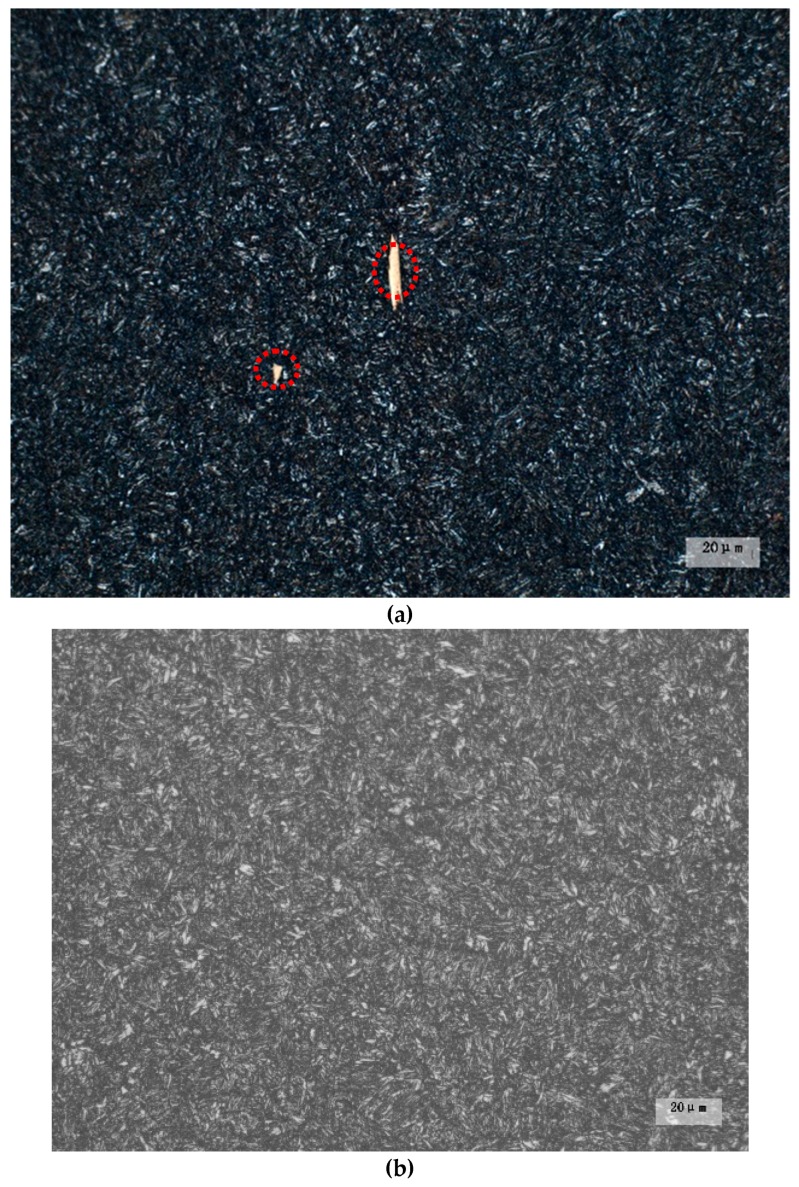
Microstructure morphology of the 50CrVA after fatigue testing: (**a**) S1#-1; (**b**) S2#-1.

The microstructure and hardness of the 50CrVA spring steel flat bar after fatigue testing are shown in Table 3. The matrix microstructure of the two materials is tempered martensite, and the hardness is almost the same. From the microstructure morphology of the 50CrVA shown in Figure 8, where the yellow secondary phase with the form of water chestnuts is visible in S1#-1, this result once again proved that the carbonitrides strongly precipitated in melt of S1#-1, and without carbonitrides, constitutional liquation occurred in S2#-1, which is consistent with the thermodynamics analysis results. Therefore, the fatigue life of the 50CrVA spring steel is mainly affected by the morphology of the secondary phase in the present work.

The longer fatigue life or good ductility of 50CrVA-S2# spring steel could be attributed to the higher solubility of V and Ti in the matrix. Further, the higher solubility of V and Ti in the matrix will result in the dispersed carbonitrides of the V and Ti elements in the 50CrVA-S2# spring steel being finer, which can significantly improve the work hardening rate of the steel by promoting the accumulation of the dislocations around the finer particles compared with the larger particles [36,37]. Based on the above results, it can be seen that thermodynamics analysis is a useful tool in determining the optimal chemical composition for spring steels. In this study, for the Fe–V–Ti–N–C microalloyed steels system, the Ti element can inhibit the matrix grain growth, the V element affects the precipitation strengthening and microstructure refinement, and the N element enhances the effect of V and Ti [38]. Therefore, one can carefully design a multiple microelements system and its contents in spring steel based on thermodynamics analysis, and then control the size, shape, and distribution of the secondary phases formed in the spring steel, thereby improving the fatigue life and toughness of high strength spring steel.

## 4. Conclusions

(1) Thermodynamics calculation results show that the solid solution contents of different elements, such as Ti, V, N, and C, decrease as the temperature decreases, especially the change of N and Ti. The change of *k*_1_, *k*_2_, *m*_1_, and *m*_2_ coefficients with temperature is more complex, and the total moles amount (*t*) of corresponding compounds increases gradually as the temperature decreases in both spring steels;
(2) For the 50CrVA-S1#, its initial precipitation temperature was 1565 °C, higher than the corresponding liquidus temperature. Carbonitrides constitutional liquation occurred, and the compound was a microparticle. For the 50CrVA-S2#, its initial precipitation temperature was 1155 °C, lower than the liquidus temperature, so no carbonitride constitutional liquation occurred, and the compound was nanoparticle;
(3) Experimental results indicate that the fatigue life reduces by about an order of magnitude when the secondary phase size changes from nanometer scale to micron level. For 50CrVA-S1#, the secondary phase size range detected in fracture place was 10.5–17 µm, and the corresponding fatigue life range was 43–63.5 × 10^3^ cycles. For the 50CrVA-S2#, the secondary phase size range detected in fracture place was 10–100 nm, and the corresponding fatigue life range was 208–242 × 10^3^ cycles.

## Figures and Tables

**Table 1:** Chemical compositions of the tested 50CrVA spring steel (wt%).

Steel	Number	C	Si	Mn	Cr	V	Ti	N	Cu	Sn	P	S
50CrVA	S1#	0.5	0.22	0.74	1.02	0.136	0.009	0.0172	0.03	0.0016	0.011	0.005
	S2#	0.49	0.26	0.75	1.01	0.15	0.002	0.0043	0.03	0.0018	0.008	0.004

**Table 2:** Interaction parameters $e_{i}^{j}$ used in the present study.

Element *j*	$e_{\mathrm{Ti}}^{j}$	$e_{V}^{j}$	$e_{N}^{j}$	$e_{C}^{j}$
C	−221/*T* − 0.072 [25]	−571.75/*T* + 0.0644 [26,27]	0.06 [28]	8890/*T* [22]
N	−19500/*T* + 8.37 [29]	−1270/*T* + 0.33 [27]	6294/*T* [22]	5790/*T* [22]
Mn	−0.043 [25]	6.2427/*T* + 0.000146 [26,27]	−8336/*T* − 27.8 + 3.652In *T* [22]	−5070/*T* [22]
Cr	−0.016 [30]	*	−65150/*T* + 24.1 [28]	−21880/*T* + 7.02 [22]
S	−0.27 [25]	−29.968/*T* [28]	0.007 [30]	0.046 [28]
P	−74.92/*T* [27]	−43.079/*T* [27]	167/*T* − 0.038 [27]	1190/*T* − 0.608 [27]
Si	177.5/*T* − 0.12 [31]	162.74/*T* − 0.0385 [26,27]	−286/*T* + 0.202 [31]	162/*T* − 0.008 [30]
Ti	212/*T* − 0.0640 [32]	30.196/*T* + 0.00313 [26,27]	−5700/*T* + 2.45 [29]	−55/*T* − 0.015 [26]
V	28.416/*T* + 0.0032 [26,27]	470/*T* − 0.22 [33]	−356/*T* + 0.0973 [34]	−134.79/*T* + 0.0185 [26,27]

* Values not found in the literature; they are assumed to be zero in current calculation.

**Table 3:** Matrix microstructure and hardness of the 50CrVA after fatigue testing.

Steels	Hardness (HRC)	Mean Hardness (HRC)	Standard Deviation	Matrix Microstructure
S1#-1	42	40.7	1.53	Tempered martensite
S1#-2	39			
S1#-3	41			
S2#-1	40.5	40.3	0.76	Tempered martensite
S2#-2	41			
S2#-3	39.5			

## References

[1] Wang Y.L., Zhuo L.C., Chen M.W., Wang Z.D. (2016). Thermodynamic model for precipitation of carbonitrides in microalloyed steels and its application on Ti-V-C-N system. Rare Met..

[2] Zhang C.L., Liu Y.Z., Jiang C. (2011). Effects of niobium and vanadium on hydrogen-induced delayed fracture in high strength spring steel. J. Iron Steel Res. Int..

[3] Htun M.S., Kyaw S.T., Lwin K.T. (2008). Effect of heat treatment on microstructures and mechanical properties of spring steel. J. Met. Mater. Miner..

[4] Yoneguchi A., Schaad J., Kurebayashi Y. (2000). Development of High Strength Spring Steel and Its Application to Automotive Coil Spring.

[5] Wu H.Z., Gao D.P., Guo H.D. (2002). Generalization of life characteristic investigation for probabilistic fatigue failure. J. Hubei Polytech. Univ..

[6] Zhang L.N., Wang P., Dong J.X., Zhang M.C. (2013). Microstructures’ effects on high temperature fatigue failure behavior of typical superalloys. Mater. Sci. Eng. A.

[7] Sun C.Q., Lei Z.Q., Xie J.J., Hong Y.S. (2013). Effects of inclusion size and stress ratio on fatigue strength for high-strength steels with fish-eye mode failure. Int. J. Fatigue.

[8] Lei Z.Q., Hong Y.S., Xie J.J., Sun C.Q., Zhao A.G. (2012). Effects of inclusion size and location on very-high-cycle fatigue behavior for high strength steels. Mater. Sci. Eng. A.

[9] Wang Q.Y., Bathias C., Kawagoishi N., Chen Q. (2002). Effect of inclusion on subsurface crack initiation and giga-cycle fatigue strength. Int. J. Fatigue.

[10] Hattori C.S., Couto A.A., Vatavuk J., deLima N.B., Reis D.A.P. (2013). Evaluation of fatigue behavior of SAE 9254 steel suspension springs manufactured by two different processes: Hot and cold winding. Experimental and Numerical Investigation of Advanced Materials and Structures.

[11] Costa L.V., Carneiro J.R.G., Catalão R.P.C., Rouhaud E. (2014). Residual stress gradients in AISI 9254 steel springs submitted to shot peening and heat treatment for increased fatigue resistance. Adv. Mater. Res..

[12] Jiang S.H., Wang H., Wu Y., Liu X.J., Chen H.H., Yao M.J., Gault B., Ponge D., Raabe D., Hirata A. (2017). Ultrastrong steel via minimal lattice misfit and high-density nanoprecipitation. Nature.

[13] Jiao Z.B., Luan J.H., Miller M.K., Liu C.T. (2015). Precipitation mechanism and mechanical properties of an ultra-high strength steel hardened by nanoscale NiAl and Cu particles. Acta Mater..

[14] Lu K. (2016). Stabilizing nanostructures in metals using grain and twin boundary architectures. Nat. Rev. Mater..

[15] Cao Y.B., Xiao F.R., Qiao G.Y., Liao B. (2014). Quantitative research on dissolving of Nb in high Nb microalloyed steels during reheating. J. Iron Steel Res. Int..

[16] Wang Y., Zhou M., Pang X., Chen X., Wang Z., Volinsky A., Tang H. (2017). Applications and Thermodynamic Analysis of Equilibrium Solution for Secondary Phases in Ti-N-C Gear Steel System with Nano-Particles. Metals.

[17] Wang C.L., Li J.S., Zhao H.M., Chen Y.F. (2009). Influence factors on solid-solution of carbonitride of niobium in steel. J. Univ. Sci. Technol. B.

[18] Andersson J.O., Helander T., Höglund L., Shi P.F., Sundman B. (2002). Thermo-Calc & DICTRA, computational tools for materials science. Calphad.

[19] Witusiewicz V.T., Bondar A.A., Hecht U., Potazhevska O.A., Velikanova T.Y. (2016). Thermodynamic modelling of the ternary B-Mo-Ti system with refined B-Mo description. J. Alloy. Compd..

[20] Drápala J., Kostiuková G., Smetana B., Madaj M., Kroupa A. (2015). Thermodynamic and experimental study of Tin-Zinc-Aluminum ternary system. Adv. Sci. Eng. Med..

[21] Uhm S., Moon J., Lee C., Yoon J., Lee B. (2004). Prediction model for the austenite grain size in the coarse grained heat affected zone of Fe-C-Mn steels: Considering the effect of in itial grain size on isothermal growth behavior. ISIJ Int..

[22] Yong Q.L. (2006). Secondary Phases in Steels.

[23] Wang Y.L., Zhou M., Pang X.L., Chen X.H., Wang Z.D., Volinsky A.A. (2018). Thermodynamic analysis of Ti_3_O_5_ nanoparticles formed in melt and their effects on ferritic steel microstructure. Materials.

[24] Wagner C. (1952). Thermodynamic of Alloys.

[25] The Japanese Society for the Promotion of Science (1988). Steelmaking Data Sourcebook. The 19th Committee on Steelmaking (Revised Edition).

[26] Morita Z., Kunisada K. (1977). Solubility of nitrogen and equilibrium of Ti-nitride forming reaction in liquid Fe-Ti alloys. ISIJ.

[27] Lee S.H., Lee K.S., Lee K.J. (2005). Evaluation of wagner interaction parameter in Fe-Mn-Si-Nb-Ti-V-C system. Mater. Sci. Forum.

[28] Chen J.X. (2010). Steelmaking Common Data Charts Manual.

[29] Morita Z., Tanaka T., Yanai T. (1987). Equilibria of nitride forming reactions in liquid iron alloys. Metall. Trans. B.

[30] Turkdogan E.T. (1996). Fundamental of Steelmaking.

[31] Evans D.B., Pehlke R.D. (1965). Equilibria of nitrogen with the refractory metals titanium, zirconium, columbium, vanadium and tantalum in liquid iron. Trans. Met. Soc. AIME.

[32] Sigworth G.K., Elliott J.F. (1974). The thermodynamics of liquid dilute iron alloys. Met. Sci..

[33] Cha W.Y., Miki T., Sasaki Y., Hino M. (2008). Temperature dependence of Ti deoxidation equilibrium of liquid iron in coexistence with Ti_3_O_5_ and Ti_2_O_3_. ISIJ Int..

[34] Pak J.J., Yoo J.T., Jeong Y.S., Tae S.J., Seo S.M., Kim D.S., Lee Y.D. (2005). Thermodynamics of titanium and nitrogen in Fe-Si met. ISIJ Int..

[35] Gan Y. (2010). Practical Manual of Modern Continuous Casting Steel.

[36] Kimura Y., Inoue T., Yin F., Tsuzaki K. (2008). Inverse temperatured ependence of toughness in an ultrafine grain-structure steel. Science.

[37] Hu J., Du L.X., Wang J.J. (2013). Effect of V on intragranular ferrite nucleation of high Ti bearing steel. Scr. Mater..

[38] Song R., Ponge D., Raabe D. (2005). Mechanical properties of an ultrafine grained C-Mn steel processed by warm deformation and annealing. Acta Mater..

